# A Polyclonal Antibody Based Immunoassay Detects Seven Subtypes of Shiga Toxin 2 Produced by *Escherichia coli* in Human and Environmental Samples

**DOI:** 10.1371/journal.pone.0076368

**Published:** 2013-10-16

**Authors:** Xiaohua He, Stephanie Patfield, Robert Hnasko, Reuven Rasooly, Robert E. Mandrell

**Affiliations:** Western Regional Research Center, Agricultural Research Service, United States Department of Agriculture (USDA), Albany, California, United States of America; Cornell University, United States of America

## Abstract

**Background:**

Shiga toxin-producing *Escherichia coli* (STEC) are frequent causes of severe human diseases ranging from diarrhea to hemolytic uremic syndrome. The existing strategy for detection of STEC relies on the unique sorbitol-negative fermentation property of the O157 strains, the most commonly identified serotype has been *E. coli* O157. It is becoming increasingly evident, however, that numerous non-O157 STEC serotypes also cause outbreaks and severe illnesses. It is necessary to have new methods that are capable of detecting all STEC strains.

**Methods and Findings:**

Here we describe the development of a sandwich ELISA assay for detecting both O157 and non-O157 STECs by incorporating a novel polyclonal antibody (pAb) against Stx2. The newly established immunoassay was capable of detecting Stx2a spiked in environmental samples with a limit of detection between 10 and 100 pg/mL in soil and between 100 and 500 pg/mL in feces. When applied to 36 bacterial strains isolated from human and environmental samples, this assay detected Stx2 in all strains that were confirmed to be *stx2*-positive by real-time PCR, demonstrating a 100% sensitivity and specificity.

**Conclusions:**

The sandwich ELISA developed in this study will enable any competent laboratory to identify and characterize Stx2-producing O157 and non-O157 strains in human and environmental samples, resulting in rapid diagnosis and patient care. The results of epitope mapping from this study will be useful for further development of a peptide-based antibody and vaccine.

## Introduction

Shiga toxin-producing *Escherichia coli* (STEC) are a group of food-borne bacteria associated with outbreaks worldwide. They cause human illnesses ranging from common diarrhea to hemolytic-uremic syndrome (HUS), a life-threatening complication [1], [2]. Ruminants are the major known reservoir of STEC [3]. Consumption of undercooked food contaminated with animal feces is the most common means of infection [4]. Various virulence factors are involved in STEC pathogenesis, and Shiga toxins (Stxs) are the most important factors [5]. There are two types of Stxs produced by STEC strains, Stx1 and Stx2, and they consist of a similar structure, an A-subunit associated with five identical B-subunits. The A-subunit is an enzymatically active *N*-glycosidase that inhibits protein synthesis by cleavage of an adenine base from the 28S rRNA component of the eukaryotic ribosomal 60S subunit, resulting in cell death [6]. The B-subunit recognizes receptors on target cells and binds to them, leading to the internalization of the toxin [7]. Despite their structural similarity, Stx1 and Stx2 exhibit significant differences in biological activity. Epidemiological and molecular typing studies indicate that STEC strains expressing Stx2 have been associated more closely with severe HUS and hemorrhagic colitis (HC) than STEC producing Stx1 or Stx1 and Stx2 [5], [8], [9]. Strains expressing both Stx1 and Stx2 are less toxic than those expressing only Stx2. This is presumably due to competition for the same host cell receptors [9]. Previous assays also showed that Stx2 was 100 times more toxic to mice than Stx1 [10], [11]. In contrast to Stx1, more subtypes have been identified for Stx2. To date, there are seven predominant Stx2 subtypes (a through g) [12], all of which have been associated with human illnesses [9], [13], [14]. The existing strategy for diagnosis of clinical samples relies on biochemical markers based on the unique sorbitol negative fermentation and ß-D-glucuronidase-positive properties of the O157 strains [15], [16], the most commonly identified STEC serotype from patients who have HUS and HC has been *E. coli* O157: H7. As more laboratories start to apply assays for the Stxs, however, more illnesses linked to non-O157 STEC serotypes are uncovered. In a report published in 2012, the big six non-O157 strains were revealed to be responsible for 113,000 illnesses annually in the United States, almost double the amount of the illnesses caused by *E. coli* O157 [17] and some cases of non-O157 illness appear to be as severe as cases associated with O157 [18]. As a result, the food industry has been required by USDA-FSIS to implement routine verification testing for the six additional non-O157 STECs in raw beef manufacturing trimmings since June 4, 2012. To comply with this policy and minimize infections, new methods that detect all STEC strains are needed.

Substantial progress has been made in the development of detection assays for STEC strains based on the presence of Stxs. However, the sensitivity and specificity of these assays for detection of Stxs present in human or environmental samples has not been validated. The Vero cell assay has been the gold standard for the detection of Stxs, but as with all cell-based activity assays, it is time-consuming, labor intensive, and requires cell culture facilities. Furthermore, a subsequent antibody-based neutralization bioassay is required in order to confirm the presence of the toxin. Stx-specific PCR assays are specific and less time-consuming, but they detect the toxin gene sequence, not the toxin itself. Immunoassays have been popular because they are simple, fast, and cost-effective. Currently, four FDA-approved immunoassays are available commercially in the United States including the ProSpecT STEC Microplate Assay (Remel Inc., Lenexa, KS), Premier EHEC (Meridian Bioscience Inc., Cincinati, OH), the Immunocard STAT!EHEC (Meridian Bioscience Inc., Cincinati, OH) and the Duopath Verotoxins Gold Labeled Immunosorbent Assay (Merck, Germany). These are ELISA-based assays. Multiple studies showed that these commercial kits often fail to detect a subset of STEC strains for unknown reasons [19], [20], [21], possibly in part due to their inability to detect certain subtypes of Stxs [2], [22]. A number of kits have not been subjected to a full evaluation, which includes testing for all known Stx subtypes. Some commercial tests give high percentage of false-positive STEC results when other pathogens are present [23].

To address these problems, we developed an immunoassay for rapid and sensitive detection of all subtypes of Stx2 by incorporating a novel pAb. We focused our study on the Stx2-producing STEC strains because they are more closely associated with the development of HUS in humans. We demonstrate that the newly established assay was capable of detecting very low amounts of Stx2 present in soil and cow feces and also validated the assay by applying it to 36 O157 and non-O157 STEC stains isolated from environmental and human samples.

## Materials and Methods

### Stx and monoclonal antibodies (mAbs)

Pure Stx1 was purchased from List Biological Laboratories, Inc. (Campbell, CA). Stx2a was purified from culture supernatant of bacterial strain RM10638 and prepared as described previously [24]. Other Stx2 subtypes were also purified from culture supernatants as described previously [25]. The mAbs against the Stx2 A-subunit and B-subunit, designated Sifin2A (VT135/6-B9) and Sifin2B (VT136/8-H4), respectively, were purchased from Sifin Institute (Berlin, Germany).

### Production and purification of rabbit polyclonal antibodies against Stx2

Stx2a recombinant toxoid was prepared as described previously [26]. The pAbs against the Stx2a toxoid were produced by Pacific Immunology Corp (Ramona, CA). Briefly, the toxoid was emulsified with either Complete Freund's adjuvant (1^st^ immunization), or incomplete adjuvant (2^nd^ to 4^th^ boosts) prior to immunization. The emulsion was injected to two rabbits, 5153 and 5154, at 3-week intervals (∼300 μg toxoid was injected per rabbit at each time point). Following the 3^rd^ injection, bleeds were collected and evaluated for anti-antigen activity by ELISA. Antibodies were purified by affinity chromatography on Protein-A conjugated agarose (Pierce, Rockford, IL) and bound antibodies were eluted with 0.1 M glycine-HCl, pH 2.7. Protein concentrations were determined with the BCA Protein Assay Kit (Pierce). The attachment of horse radish peroxidase (HRP) to antibodies was performed using a Lightning-Link HRP Conjugation Kit (Innova Biosciences, Cambridge, UK).

### Peptide mapping of the pAb

Type 2 Epitope Mapping Service was ordered from PEPperPRINT (Heidelberg, Germany). The Stx2a protein sequence was translated into 10, 12, and 15mer peptides with a peptide-peptide overlap of 9, 11, and 14 amino acids and printed as duplicates on PEPperCHIP. Protein A purified pAb IgG (10 µg) was used for the immunoassay.

### Preparation of bacterial culture supernatant containing Stx2

Pure bacterial culture supernatants were prepared from the strains listed in Table 1 and Table 2. The cells were grown overnight in Luria-Bertani (LB) liquid medium at 37°C to an optical density at 600 nm of approximately 1.8. Following centrifugation at 13,000×*g* for 10 min at 4°C, the supernatants were collected and filtered through a 0.2 µm filter to remove intact cells and other debris.

**Table 1:** Detection of Stx2 in culture supernatants of STEC strains.

			This study		PremierEHEC	
Strain	Serotype	Stx2 subtype^a^	s/n^b^ ± SD	Result^c^	s/n^b^ ± SD	Result^c^
RM4876	O157: H7	Stx-negative	1±0	−	1±0	−
RM10638	O157: H7	Stx2a	162±2	+	50±7	+
RM7005	O118: H12	Stx2b	11±1	+	3±1	−
RM10058	O157: H7	Stx2c	51±3	+	51±8	+
RM8013	ND	Stx2d	40±1	+	50±8	+
RM7110	O139: NM	Stx2e	7±1	+	2±1	−
RM7007	O128: H2	Stx2f	12±1	+	48±8	+
RM10468	ND	Stx2g	28±1	+	2±1	−

a Stx2 subtype was determined by PCR method described previously [24]; ^b^ Signal to noise; ^c^ Results were considered Stx2-positive when signal to noise ratio >3 and Stx2-negative when signal to noise ratio ≤3; ND indicates not determined; SD indicates standard deviation.

**Table 2:** Results of PCR and ELISA for detection of Stx2 in *E. coli* strains isolated from human and environmental samples.

Strain	Serotype	Origin	PCR^a^		ELISA^b^	Strain	Serotype	Origin	PCR^a^		ELISA^b^
			*stx1*	*stx2*	Stx2				*stx1*	*stx2*	Stx2
RM1239	O157: H7	Human	−	+	+	RM9322	O111	Water	+	−	−
RM1913	O157: H7	Human	−	+	+	RM9907	O111	Feral Pig	+	−	−
RM2367	O157: H7	Human	+	+	+	RM9975	O111	Crow	+	−	−
RM6649	O157: H7	Human	+	+	+	RM12788	O111	Human	+	+	+
RM7543	O157: H7	Human	+	+	+	RM7783	O113	Crow	−	+	+
RM7375	O26	Human	+	−	−	RM7788	O113	Water	−	+	+
RM7927	O26	Water	+	−	−	RM7958	O113	Cow feces	+	+	+
RM8426	O26	Water	+	−	−	RM10466	O113	Cow feces	−	+	+
RM10817	O26	Cow feces	+	−	−	RM10940	O113	Cow feces	−	+	+
RM13151	O26	Human	+	−	−	RM5856	O121	Human	−	+	+
RM9413	O45	Cow feces	+	−	−	RM6848	O121	Lettuce	−	+	+
RM13506	O45	Human	+	−	−	RM8082	O121	Cow feces	+	−	−
RM13752	O45	Cow feces	+	−	−	RM8352	O121	Sediment	−	+	+
RM9882	O103	Cow feces	+	−	−	RM8876	O145	Water	+	−	−
RM10061	O103	Feral Pig	+	−	−	RM9306	O145	Cow feces	+	−	−
RM10408	O103	Crow	+	−	−	RM9872	O145	Cow feces	−	+	+
RM13508	O103	Human	+	−	−	RM9917	O145	Feral Pig	+	−	−
RM7370	O111	Water	+	+	+	RM12238	O145	Human	−	+	+

a PCR was performed as described previously [27]. ^b^ ELISA results were considered Stx2-positive when signal to noise ratio >3 and Stx2-negative when signal to noise ratio ≤3.

To test the matrix effect from environmental samples on the ELISA performance, samples of STEC-negative soil and feces (10 g) were transferred into 250-mL sterile flasks containing 90 mL tryptic soy broth (TSB) and incubated for 2 h at 25°C and then 8 h at 42°C with shaking. For STEC-negative control, 99 mL of water was mixed with 11 mL of 10 X TSB and incubated at the same conditions. The enriched broth was filter-sterilized and aliquots of the broth were spiked with serial dilutions of Stx2 and analyzed by ELISA.

### ELISA

ELISAs were performed as described previously [26]. For the capture ELISA, the pAb was used as a capture antibody (1 µg/mL); pure or crude Stx2 in bacterial culture supernatant (100 µL/well) was used as the antigen; the HRP- conjugated pAb was used as a detector (100 ng/mL). HRP activity was monitored using the SuperSignal West Pico Chemiluminescent Substrate (Pierce). The luminescent counts (counts per second) were measured with a plate reader (Perkin-Elmer, Shelton, CT). For the direct ELISA, the assay was performed similarly to the capture ELISA except that the plate was directly coated with antigens without pre-coating a capture antibody. The Limit of Detection (LOD) was defined as the lowest toxin concentration at which the average ELISA reading was three standard deviations above the negative control.

### Polyacrylamide gel electrophoresis (PAGE) and Western blot

All gel electrophoresis equipment, buffers, gels and PVDF membranes were purchased from Invitrogen (Carlsbad, CA). The Stx2a toxoid was separated by SDS-PAGE using 4–12% NuPAGE (denatured) Novex Bis-Tris mini gels following the manufacturer's protocol. To visualize proteins directly after gel electrophoresis, 1 µg of toxoid was loaded in each lane and gels were stained with Coomassie Blue G-250 (Bio-Rad, Hercules, CA). For western blot analysis, 0.5 µg of toxoid was loaded and separated by PAGE. Proteins were electrotransferred to PVDF membranes (0.45 µm). The membranes were blocked with 2% Amersham ECL Prime Blocking Reagent (GE Healthcare, UK), then probed with a mixture of mAbs (Sifin2A and Sifin2B) against the Stx2 A- and B-subunits (20 µg/mL) or rabbit 5154 IgG (20 µg/mL), followed by goat anti-mouse or rabbit IgG-HRP (Promega, Madison, WI, 1∶500,000). Bound antibody was detected using the Amersham ECL-Plus Western Blotting Detection System (GE Healthcare) according to the manufacturer's protocol.

### Antibody-antigen binding affinity measurements

Real-time binding assays between purified antibodies and purified Stx2a protein were performed as described previously [26] using biolayer interferometry with an Octet QK system (Forte-bio, Menlo Park, CA). Briefly, the biotinylated pAb was coupled to streptavidin biosensors (Forte-bio) at 10 µg/mL in phosphate-buffered saline (PBS, pH 7.2). Probes coupled to antibody were allowed to bind to Stx2a at seven different concentrations ranging from 2 to 142 nM. Binding kinetics were calculated using the Octet QK software package (Data Acquisition 7.0).

### Neutralization of Stx2a mediated cytotoxicity in Vero cells

Fresh Vero cells were seeded on 96-well plates at 1×10^5^ cells/ml (100 µL/well) overnight in Dulbecco's Modified Eagle Medium (DMEM, Invitrogen) supplemented with 10% fetal calf serum (Invitrogen) and incubated in a humidified incubator (37°C, 5% CO_2_). Cells treated with a combination of Stx2a (10 ng/mL) and pAb were first incubated at 4°C for 1 hour, then shifted to 37°C overnight. The cytotoxicity was assessed using CellTiter-Glo reagent (Promega) according to the manufacturer's instruction, except that the reagent was diluted 1∶5 in PBS prior to use. Luminescence was measured on a Victor 3 plate reader (Perkin Elmer). All treatments were performed in triplicate. Cells grown in medium without toxin and pAb were used as a negative control (0% toxicity). The cytotoxicity for cells was calculated as follows: [(cps from negative control – cps from samples treated)/cps from negative control] ×100. The relative cytotoxicity after neutralization was calculated by normalizing the toxicity of Stx2 without neutralization by pAb as 100%.

## Results

### Development and characterization of a polyclonal antibody against Stx2

**Figure 1:**
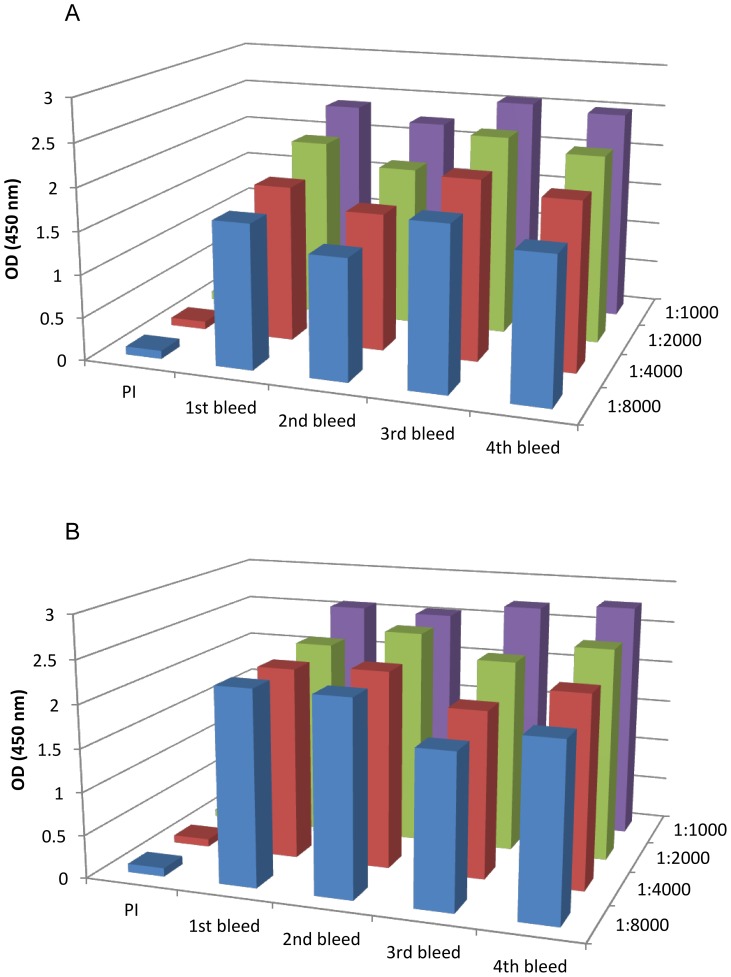
Activity of antiserum from pre-immune (PI) rabbits, 1^st^ bleeding (one week after 3^rd^ immunization), 2^nd^ bleeding (two weeks after 3rd immunization), 3^rd^ bleeding (one week after boost with additional immunogen), and 4^th^ bleeding (three weeks after boost). ELISA was performed using Sifin 2B as a capture antibody (1 μg/mL), Stx2 toxoid (10 ng/mL), and antisera of rabbits 5153 (**A**) and 5154 (**B**) diluted in a range of 1∶1,000–1∶8,000. Each column represents the mean of 3 independent repeats performed in duplicates, the standard deviation for each column ranges from 0.01 to 0.15.

To develop a sensitive and specific immunoassay, we started our work by generation of novel antibodies. To produce a pAb that binds specifically to the native Stx2, a recombinant non-catalytically active Stx2a was generated by changing the toxin active site, glutamic acid (E) at position 167 of the A subunit to glutamine (Q) [26]. The two rabbits immunized with the Stx2a (E167Q) recombinant toxoid showed high antibody serum titers (≥1∶8000), as indicated in Figure 1. The 1^st^, 2^nd^, 3^rd^, and 4^th^ bleed of Rabbit 5154 (Figure 1B) were pooled and used to purify IgG for further experimentation.

**Figure 2:**
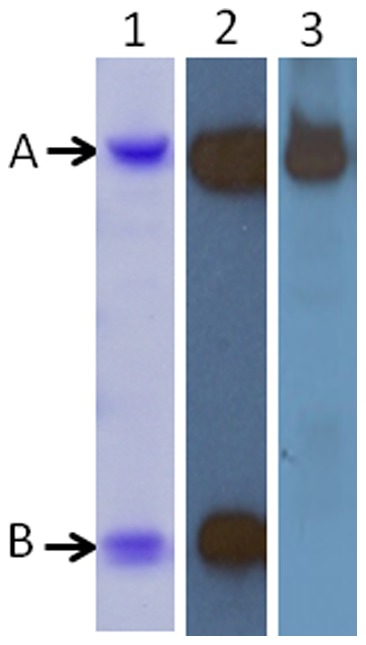
Reactivity of immunized rabbit serum IgG to Stx2a. Lane 1, Coomassie stained SDS-PAGE with 1 μg of purified Stx2a; lanes 2 and 3, Western blot of 0.5 μg of purified Stx2a probed with a mixture of mouse mAbs against Stx2 A- and B-subunits and rabbit 5154 serum IgG followed by horseradish peroxidase-conjugated goat secondary antibodies against mouse or rabbit IgG. The A and B subunit positions are indicated by arrows.

The pAb obtained bound to both the recombinant Stx2a toxoid and the wild type Stx2a but did not react with Stx1 when tested by direct ELISA (Table 3), indicating the pAb is Stx2-specific. Although the toxoid injected into the rabbits contained both the A and B subunits, the majority of antibodies in the pAb pool produced by the rabbit bound to the A subunit (Figure 2, lane 3) in SDS-PAGE/Western blotting analyses and very weak signals were found at the predicted B-subunit position in films with extended exposure time (data not shown). The presence of both the A and B subunits of the toxin was verified by Coomassie staining of the toxoid on the SDS-PAGE (Figure 2, lane 1) and Western blot probed with a mixture of mAbs against the Stx2 A and B subunits (Figure 2, lane 2).

**Table 3:** Toxin specificity of the polyclonal antibody tested by direct ELISA.

Toxin	μg/mL	Luminescent counts	SD
PBS Control	0	213	21
Stx1	1	277	51
Stx2a	1	1468613	54406

**Figure 3:**
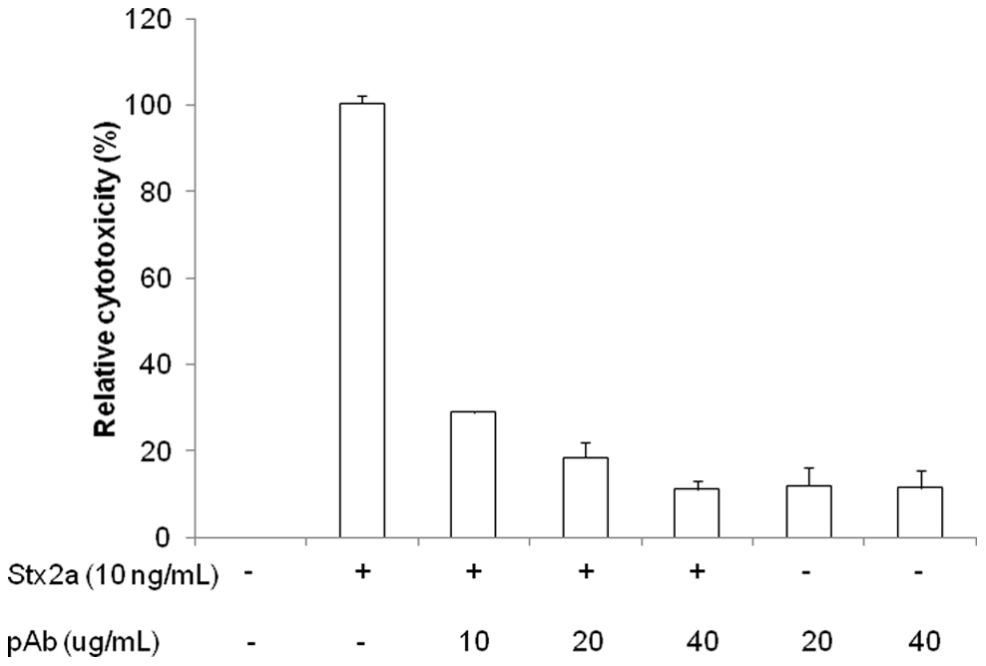
Neutralization of Stx2a cytotoxicity with the pAb. Vero cells were incubated in DMEM medium containing Stx2a (10 ng/mL) with or without the presence of the pAb. The cytotoxicity to cells was calculated as [(cps from negative control – cps from samples treated)/cps from negative control] ×100. The luminescent counts from cells grown in DMEM medium were used as a negative control. The relative cytotoxicity was calculated by normalizing each value to the cytotoxicity of Stx2a without adding pAb as 100%. The results represent the mean ± SD of three replicates from one representative experiment. Three individual experiments were performed.

To quantitate the affinity of the pAb for Stx2a protein, we used biolayer interferometry to examine the pAb binding to purified Stx2a. A dissociation constant of 1.16 nM was obtained, indicating that the pAb can bind Stx2a with high affinity. The high affinity of the pAb to Stx2a led us to test its potential to neutralize Stx2a cytotoxicity. It was found that the pAb was able to neutralize 10 ng/mL of Stx2a in a dose-dependent manner in Vero cells. When the concentration of pAb was increased to 40 µg/mL, Stx2a cytotoxicity was reduced to 10%, similar to the value observed for control cells treated with the pAb without any toxin (Figure 3). The 10% residual toxicity could be caused by some components in the rabbit serum. The ability to induce toxin-neutralizing antibodies in rabbits suggested that the toxoid used in this study could be a good candidate for vaccine development.

### Development of a new immunoassay for all subtypes of Stx2

**Figure 4:**
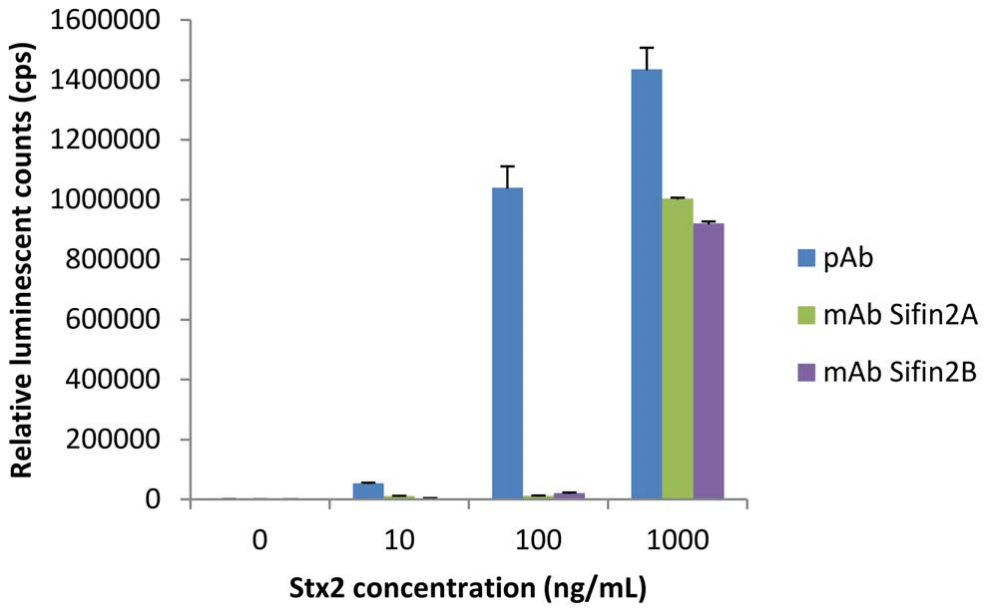
Comparison of antigen binding capacity between pAb derived from RB 5154 and commercially available mAbs, Sifin2A and Sifin2B. Coating antigen (Stx2a) was diluted in PBS at concentrations indicated. Primary antibodies (pAb or mAb) were used at 1 µg/mL and secondary antibodies (goat anti-rabbit or goat anti-mouse IgG) conjugated with HRP were used at 1∶5,000. Each bar represents the mean of three independent repeats performed in duplicates.

**Figure 5:**
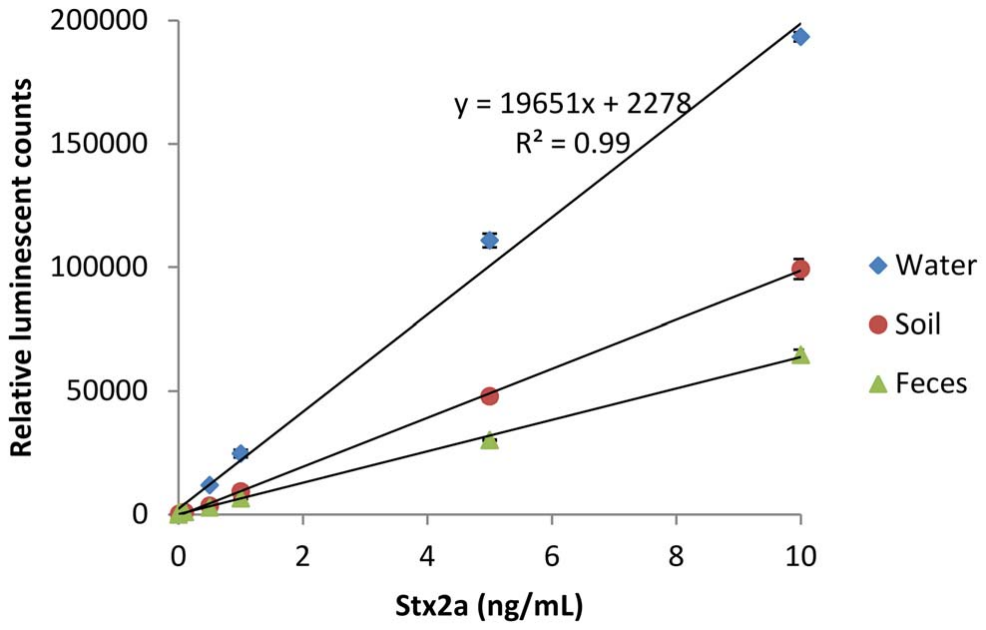
Detection of Stx2a spiked in water (♦) and broth of soil (•) and feces (▴) by a sandwich ELISA using Stx2-pAb as capture antibody (1 µg/mL) and HRP conjugated Stx2-pAb as detector antibody (100 ng/mL). Data represent the average of three determinations ± SD.

The binding efficiency of our newly developed pAb to Stx2a was compared with two commercial mAbs, Sifin2A and Sifin2B, in a direct ELISA format. As shown in Figure 4, the pAb gave much higher signals than these two mAbs at various concentrations, indicating its high affinity to Stx2a directly coated on the plate. We then assembled a sandwich ELISA using this same pAb for both capture and detection. A linear standard curve with R^2^ = 0.99 was observed within the range of 10 to 10,000 pg/mL (Figure 5). The LOD for Stx2a in water was between 10 and 100 pg/mL (the average ELISA reading at 10 pg/mL = 357, at 100 pg/mL = 1193, Bkg +3SD  = 720). We next tested the capability of the sandwich ELISA for detecting other subtypes of Stx2. Because pure toxin preparations are not available for all subtypes, culture supernatants from bacterial strains expressing Stx2a through Stx2g were collected and analyzed. Culture supernatants of *E. coli* O157: H7 strain RM4876 (which does not express Stx) was used as a negative control. Our results indicate that the sandwich ELISA developed in this study was able to detect all subtypes of Stx2 (Table 1), and the bacterial supernatant containing Stx2a gave the highest signal to noise ratio. This could be because the cumulative pool of the Stx2a pAbs favors binding epitopes in Stx2a over the other subtypes or the amount of Stx2a present in the bacterial supernatant is more abundant than other subtypes.

### Effects of environmental sample matrices on the sandwich ELISA performance

To determine if the sandwich ELISA could be used for detection of Stx2 produced in environmental samples, samples of soil and cow feces were collected and enriched in TSB broth (to simulate the processes used for isolation of STEC strains). The TSB broth was pre-tested to be STEC-negative by PCR and culture methods [27] and then spiked with pure Stx2a. Three samples from each matrix (cow feces and soil) were collected from different animals and/or different locations in Salinas, California and pooled before testing. Our results indicate that Stx2a could be detected in these matrices (Figure 5) and the LOD in soil is between 10 and 100 pg/mL (the average ELISA reading at 10 pg/mL = 237, at 100 pg/mL = 903, Bkg +3SD  = 393).; in feces is between 100 and 500 pg/mL (the average ELISA reading at 100 pg/mL = 1003, at 500 pg/mL = 2867, Bkg +3SD  = 1431). Although the LOD for Stx2a in soil is the same as that in water, the signal to noise ratio obtained from soil samples was significantly lower than that from sterilized water (P<0.05), especially when the concentrations of Stx2a was higher than 0.5 ng/mL, suggesting the presence of the matrix effect and the inhibitory effect from feces is higher than that from soil. Sensitivity of the ELISA may be improved by optimizing sample preparation steps to remove inhibitory components from these samples or by enriching Stxs in the STEC-positive samples using an enrichment medium.

### Application of the ELISA for identifying STEC strains isolated from environmental and human samples

We next evaluated the sandwich ELISA in a blind study with 36 STEC strains isolated from human and environmental samples. Although the Stx subtype carried by each bacterium strain was not known, the presence of *stx1* and *stx2* genes was confirmed by a PCR method described previously using Qiagen multiplex PCR master mix and a Tetrad thermal cycler [27]. In this study, supernatants of pure bacterial cultures were used as toxin sources for the ELISA. Table 2 demonstrates that our ELISA was capable of detecting Stx2 in all samples that were shown to be *stx2*-positive by real-time PCR and did not detect Stx2 in any samples that were shown *stx2*-negative by PCR. In addition, the assay did not cross react with Stx1 expressed by any STEC strains. These results indicate that the ELISA developed in this study had 100% sensitivity and specificity based on the results obtained from the PCR and can be applied to both O157 and non-O157 STEC strains isolated from human and environmental samples.

### Epitope mapping of the pAb

Our immunoassay data indicates that the pAb was able to detect all subtypes of Stx2, but not Stx1. To better understand the available antigenic sites in our Stx2a toxoid immunogen, we performed epitope mapping using the pAb and a peptide array containing 10, 12, and 15mer Stx2a-derived liner peptides with a peptide-peptide overlap of 9, 11, and 14 amino acids. Table 4 and 5 shows the consensus amino acid sequences (underlined) identified using overlapping Stx2a peptides (Accession # AAM70029) within the Stx2a A- and B- subunit primary structures, respectively. The corresponding amino acid sequences for Stx2b through Stx2g and Stx1 are shown following sequence alignment (NCBI, COBALT). Mismatched amino acids in Stx2b-g and Stx1 relative to Stx2a peptides are highlighted in grey. This analysis shows the high degree of amino acid mismatch in the Stx1 sequence at the motifs corresponding to Stx2-pAb binding epitopes and is consistent with the selectivity of our pAb to Stx2 subtypes. All of the Stx2 subtypes each share at least one identical binding motif with those defined by our peptide mapping. The RISNVLPEY epitope at Stx2a A-subunit 204–212aa is identical in all Stx2 subtypes with one YF substitution in Stx2b. The impact of this favored substitution is thought to be minimal and residues NVLPE map to a surface location on the 3-D structure of Stx2a (Figure S1; panel A). Two consensus pAb binding peptide sequences corresponding to the B-subunit (GKIEFSK and LQSAQLT) are 100% conserved in all seven Stx2 subtypes. The GKIEFSK peptide (Stx2a B-subunit 6–12aa) presents a structure mapping to a large surface area of the B-subunit protein (Figure S1; panel B) that would be available for antibody binding. The LQSAQLT peptide (St2a B-subunit 39–45aa), however, maps to the inner ring of the B-subunit pentamer (Figure S1; panel C) and in the presence of the A-subunit, antibody binding to this epitope might be minimized (Figure S1; panel D). Although the DTFTV peptide (Stx2a B-subunit residues 17–21aa) is identical in Stx1 B-subunit, the lack of pAb binding to Stx1 argues that these epitopes must differ structurally between the two B-subunits. One possible explanation is found in the surrounding sequence of residues (Stx2a B-subunit residues 1–31aa) where Stx1 shows considerable divergence with 13 mismatched amino acids including an added proline.

**Table 4:** Stx2 polyclonal antisera binding to consensus epitopes of A-subunit determined using overlapping Stx2a peptides.

Accession#	Toxin	aa * 29–38	76–86	144–149	188–200	204–212	252–256	262–265	289–295
AAM70029	Stx2a	EHISQGTTSV	LYVAGFVNTAT	EFSGNT	VYTMTPGDVDLTL	RISNVLPEY	AVNEE	QITG	SQFLYTT
AAD12174	Stx2b	EHISQGTTSV	LYVAGFVNTAT	EFSGNA	VYTMTPEEVDLTL	RISNVLPEF	SVNEE	QITG	AHSLNTS
AAA19623	Stx2c	EHISQGTTSV	LYVAGFVNTAT	EFSGNT	VYTMTPGDVDLTL	RISNVLPEY	AVNEE	QITG	SQFLYTT
AAK66972	Stx2d	EHISQGTTSV	LYVAGFVNTAT	EFSGNT	VYTMTPGDVDLTL	RISNVLPEY	AVNEE	QITG	SQSLYTT
AAA19189	Stx2e	EHISQGATSV	LYVAGFVNTTT	EFSGNT	VYTMTPGDVDLTL	RISNVLPEY	AVNEE	QITG	SQSLYTT
CAB64953	Stx2f	GNISQGGVSV	LYVAGFINTET	EFRGRS	LYTMTAQDVDLTL	RISNVLPEY	SVSQK	QIVG	PQDLTEP
AAP37403	Stx2g	EHISQGATSV	LYVAGFVNTAT	EFSGNT	VYTMTPEDVDLTL	RISNVLPEY	YVNEE	QISG	SQSLYTT
BAC78637	Stx1	QTISSGGTSL	LYVTGFVNRTN	SHSGTS	SYVMTAEDVDLTL	RLSSVLPDY	MASDE	PADG	TI–

* Amino acid position is based on the protein sequence of Stx2a subtype.

Highlighted fonts in alignment indicate divergent amino acids within defined epitope.

Accession # indicates the toxin protein accession number in GenBank. Information regarding the proteins can be found at http://www.ncbi.nlm.nih.gov/protein.

**Table 5:** Stx2 polyclonal antisera binding to consensus epitopes of B-subunit determined using overlapping Stx2a peptides.

Accession#	Toxin	aa* 6–12	17–21	39–45	59–69
AAM70030	Stx2a	GKIEFSK	DTFTV	LQSAQLT	GSGFAEVQFNN
AAD12175	Stx2b	GKIEFSK	DTFTV	LQSAQLT	GSGFAEVQFN-
AAA19624	Stx2c	GKIEFSK	DTFTV	LQSAQLT	GSGFAEVQFNN
AAK66973	Stx2d	GKIEFSK	DTFTV	LQSAQLT	GSGFAEVQFNN
AAA19190	Stx2e	GKIEFSK	NTFTV	LQSAQLT	GSGFAQVKFN-
CAB64954	Stx2f	GKIEFSK	DTFTV	LQSAQLT	GSGFAQVKFN-
AAP37404	Stx2g	GKIEFSK	NTFTV	LQSAQLT	GSGFAEVQFNN
BAC78638	Stx1	GKVEYTK	DTFTV	LLSAQIT	GGGFSEVIFR-

* Amino acid position is based on the protein sequence of Stx2a subtype.

Highlighted fonts in alignment indicate divergent amino acids within defined epitope.

Accession # indicates the toxin protein accession number in GenBank. Information regarding the proteins can be found at http://www.ncbi.nlm.nih.gov/protein.

## Discussion

With the continuous increase in outbreaks associated with non-O157 STEC strains, the conventional detection and isolation methods solely relying on the unique sorbitol negative fermentation property of the O157 strain are not sufficient, new approaches that can detect all STEC strains are needed. The purpose of this study is to develop a new method to address this new problem. Since multiple evidences indicate Stx2-producing STEC strains are more frequently associated with the development of HUS than Stx1-producing strains [5], [8], [28], we targeted our studies to the Stx2-producing STEC. To determine if a bacterial strain is a STEC, the best way is to examine if the strain produces Stxs. An immunoassay that identifies STEC strains based on the presence of Stxs would be a good approach. Currently, there are several such assays available [22], however, none of them detects all Stx2-producing STEC strains. One of the most important reasons is that the antibodies used in these assays only recognize a subset of Stx2. To develop a better assay we started with generating a new pAb because pAbs usually have higher reactivity than mAbs due to the polyvalency (multiple epitopes to react with) versus the monovalency of a mAb. When deciding what immunogens to use, we thought that toxoids prepared by conventional methods, such as heating or chemical inactivation are more likely to be distorted in structure and antibodies produced using these toxoids often react with denatured toxins instead of with biologically active toxins [29]. Therefore, we used a previously developed recombinant toxoid of Stx2a as an immunogen [26] in this study. This toxoid was created by replacing the glutamic acid at position 167 of the Stx2a A-subunit (an amino acid critical to the enzymatic activity of the toxin) with glutamine. As we expected, polyclonal antibodies yielded from this toxoid bound strongly to the native Stx2a with a dissociation constant of 1.16 nM and were Stx2-specific. Most antibodies in the pAb pool bound to the A-subunit, suggesting that the Stx2 B-subunit is less immunogenic compared to the A-subunit, which is consistent with the results we observed in mice [26].

In order to develop a new assay that detects all subtypes of Stx2 produced by *E. coli*, we used the identical pAb, non-modified as capture antibody and HRP-conjugated as detection antibody. This approach is advantageous because it not only shortens the assay time by omitting using a secondary antibody, but also minimizes cross-reactivity and nonspecific interaction from different antibodies. To examine the performance of the assay, we directly compared our assay with the commercial Meridian Premier EHEC assay using the same set of samples (culture supernatants from bacterial strains expressing Stx2a through Stx2g). The Premier EHEC was chosen because it is one of the most well known commercial assays for Stxs on the market. The current USDA-FSIS protocol for detection of STEC O157 uses it to confirm the presence of Stxs. Our results indicate that the ELISA developed in this study was capable of detecting all subtypes of Stx2, while the Premier EHEC immunoassay detected subtypes of Stx2a, Stx2c, Stx2d, and Stx2f but failed to detect Stx2b, Stx2e, and Stx2g (Table 1). Also the sensitivity of our ELISA for most subtypes (Stx2a, 2b, 2e, and 2g) was much better than that of the Premier EHEC assay based on the signal to noise ratio data (Table 1). The presence of Stx2 in all bacterial culture supernatants was pre-confirmed by Vero cell assays (data not shown). The Premier EHEC test utilizes a mAb as a capture, a polyclonal antibody as a detector, and an enzyme conjugated secondary antibody for color development. Based on our observations, very few, if any, mAbs are able to react with all subtypes of Stx2. The downside of using a pAb-based approach is that there may be a batch to batch (rabbit to rabbit) variation in immune response to the Stx2, although no significant variation was observed between the pAbs produced by two rabbits in our experiments.

In summary, we have developed a simple and unique immunoassay for detecting all seven subtypes of Stx2 by incorporating a novel pAb in the assay. Despite the matrix effect present in samples, Stx2a was easily detected when its levels were above 10 pg/mL in soil and 100 pg/mL in feces, indicating the potential use of this ELISA for identifying STEC strains from real environmental samples based on the presence of Stx2. But we must point out that the current in-house sandwich ELISA can detect Stx2a only in STEC-positive soil or cow feces samples after enrichment in the TSB medium supplemented with mitomycin C (50 ng/mL) for 24 hours at 37°C, it cannot detect the Stxs directly from environmental samples without enrichment (unpublished data). Application of this assay to 36 STEC strains isolated from human and environmental samples demonstrates a 100% sensitivity and specificity. This assay will be useful for solving current problems facing the food industry and regulatory agencies and provide a valuable tool for incorporation in HACCP systems.

## Supporting Information

Figure S1 In silico mapping of Stx2-pAb epitopes to the 3-D structure of Stx2a A/B-subunits. The structure of Stx2a was obtained using protein data base entry 1r4p (8) and CPK representation of A-subunit (blue) and the B-subunit pentamer (green) were rendered in Molsoft ICM-Browser. Stx2a-pAb binding to consensus amino acid sequences was determined using overlapping peptides corresponding to Stx2a A/B-subunit sequences (Accession # AAM70029). Panel-A shows the spatial location of the consensus sequence RISNVLPEY in red mapped to the Stx2a A-subunit. Panel-B depict the spatial location of consensus sequences GKIEFSK and panels C-D shows the location of LQSAQLT residues in red mapped to Stx2a B-subunits.
(TIF)
