# Supplementary figures and images for: A Polyclonal Antibody Based Immunoassay Detects Seven Subtypes of Shiga Toxin 2 Produced by Escherichia coli in Human and Environmental Samples
Source: PLoS One. 2013 Oct 16;8(10):e76368. doi: 10.1371/journal.pone.0076368 (PMC3797811; doi:10.1371/journal.pone.0076368)

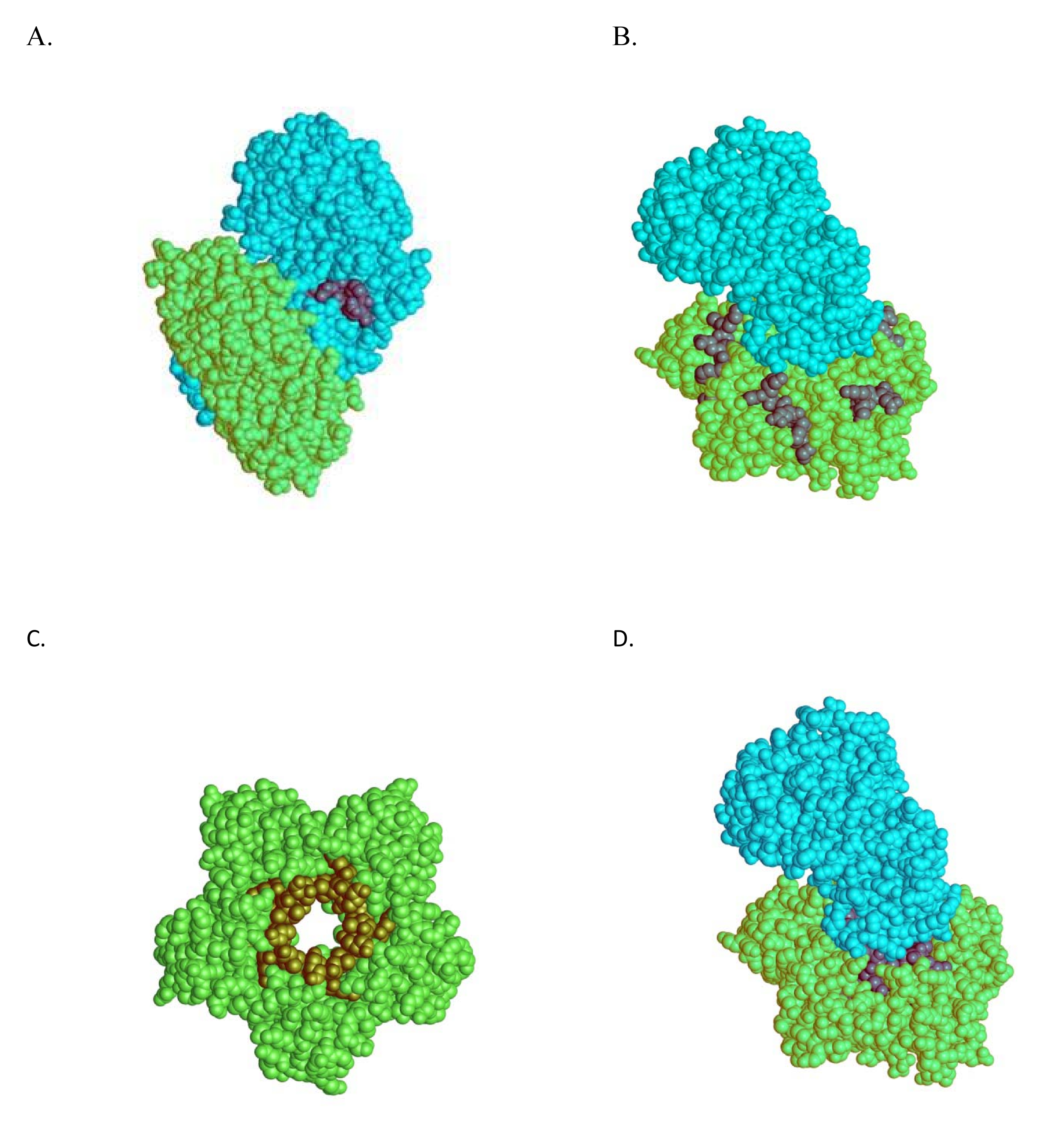

Supplement: Figure S1 — In silico mapping of Stx2-pAb epitopes to the 3-D structure of Stx2a A/B-subunits. The structure of Stx2a was obtained using protein data base entry 1r4p (8) and CPK representation of A-subunit (blue) and the B-subunit pentamer (green) were rendered in Molsoft ICM-Browser. Stx2a-pAb binding to consensus amino acid sequences was determined using overlapping peptides corresponding to Stx2a A/B-subunit sequences (Accession # AAM70029). Panel-A shows the spatial location of the consensus sequence RISNVLPEY in red mapped to the Stx2a A-subunit. Panel-B depict the spatial location of consensus sequences GKIEFSK and panels C-D shows the location of LQSAQLT residues in red mapped to Stx2a B-subunits. (TIF) [file pone.0076368.s001.tif]
